# Potential proton and photon dose degradation in advanced head and neck cancer patients by intratherapy changes

**DOI:** 10.1002/acm2.12189

**Published:** 2017-09-18

**Authors:** Kristin Stützer, Annika Jakobi, Anna Bandurska‐Luque, Steffen Barczyk, Carolin Arnsmeyer, Steffen Löck, Christian Richter

**Affiliations:** ^1^ OncoRay – National Center for Radiation Research in Oncology Faculty of Medicine and University Hospital Carl Gustav Carus Technische Universität Dresden Helmholtz‐Zentrum Dresden ‐ Rossendorf, Fetscherstr. 74, PF 41 01307 Dresden Germany; ^2^ Helmholtz‐Zentrum Dresden ‐ Rossendorf Institute of Radiooncology – OncoRay Bautzner Landstr. 400 01328 Dresden Germany; ^3^ Department of Radiotherapy and Radiation Oncology Faculty of Medicine and University Hospital Carl Gustav Carus Technische Universität Dresden Fetscherstr. 74 01307 Dresden Germany; ^4^ German Cancer Consortium (DKTK), partner site Dresden Germany and German Cancer Research Center (DKFZ) Im Neuenheimer Feld 280 69192 Heidelberg Germany; ^5^ Present address: Department of Radiotherapy and Radiooncology St. Agnes‐Hospital Bocholt Klinikum Westmünsterland, Barloer Weg 125 46397 Bocholt Germany

**Keywords:** dose degradation, head‐and‐neck cancer, IMPT, IMRT, interfractional changes, set‐up error

## Abstract

**Purpose:**

Evaluation of dose degradation by anatomic changes for head‐and‐neck cancer (HNC) intensity‐modulated proton therapy (IMPT) relative to intensity‐modulated photon therapy (IMRT) and identification of potential indicators for IMPT treatment plan adaptation.

**Methods:**

For 31 advanced HNC datasets, IMPT and IMRT plans were recalculated on a computed tomography scan (CT) taken after about 4 weeks of therapy. Dose parameter changes were determined for the organs at risk (OARs) spinal cord, brain stem, parotid glands, brachial plexus, and mandible, for the clinical target volume (CTV) and the healthy tissue outside planning target volume (PTV). Correlation of dose degradation with target volume changes and quality of rigid CT matching was investigated.

**Results:**

Recalculated IMPT dose distributions showed stronger degradation than the IMRT doses. OAR analysis revealed significant changes in parotid median dose (IMPT) and near maximum dose (*D*_1ml_) of spinal cord (IMPT, IMRT) and mandible (IMPT). OAR dose parameters remained lower in IMPT cases. CTV coverage (*V*_95%_) and overdose (*V*_107%_) deteriorated for IMPT plans to (93.4 ± 5.4)% and (10.6 ± 12.5)%, while those for IMRT plans remained acceptable. Recalculated plans showed similarly decreased PTV conformity, but considerable hotspots, also outside the PTV, emerged in IMPT cases. Lower CT matching quality was significantly correlated with loss of PTV conformity (IMPT, IMRT), CTV homogeneity and coverage (IMPT). Target shrinkage correlated with increased dose in brachial plexus (IMRT, IMPT), hotspot generation outside the PTV (IMPT) and lower PTV conformity (IMRT).

**Conclusions:**

The study underlines the necessity of precise positioning and monitoring of anatomy changes, especially in IMPT which might require adaptation more often. Since OAR doses remained typically below constraints, IMPT plan adaptation will be indicated by target dose degradations.

## 1 INTRODUCTION

Tumor‐conformal treatment plans with steep dose gradients are required for radiotherapeutic treatment of advanced head‐and‐neck cancer (HNC). The target volumes are surrounded by critical normal tissue structures and may comprise some hundred milliliters when including lymph nodes and/or lymphatic pathways. Conformal dose distributions are typically provided by advanced photon therapy techniques like intensity‐modulated radiotherapy (IMRT).^1^ While intensity‐modulated proton therapy (IMPT) can more effectively reduce the dose to healthy tissue^2^, ^3^, ^4^, ^5^ and thus allow for further dose escalation,^6^ proton beams are prone to range uncertainties if the penetrated tissue changes during therapy. Gradual intratherapy changes in HNC patient anatomy, mainly caused by weight loss, shrinkage of tumor, and shift of close‐by structures can be assessed via imaging, e.g. by computed tomography (CT), and are of concern during radiotherapy treatment.^7^, ^8^ The dosimetric consequences of such changes, namely the potential underdose of target volumes and overdose in organs at risk (OARs), have been quantified in detail for IMRT plans.^9^, ^10^ Treatment plan adaptation can be used to prevent severe dose degradation throughout the fractionated treatment course^11^ and is related with lower normal tissue complication probabilities.^12^ For IMRT treatment, up to two adaptation steps was reported to be sufficient and is logistically feasible.^13^, ^14^, ^15^, ^16^ Adaptive IMRT has been shown to be associated with improved locoregional control,^17^ especially for advanced tumor stages.^18^

Similar detailed clinical data are not available for IMPT treatment. Just recently, Placidi et al.^19^ reported plan adaptations for 12 of 102 HNC patients treated with proton therapy between 2007 and 2014. Since proton range is sensitive to tissue changes, positioning uncertainties, and interfractional anatomy changes, stronger dose degradations are possible. First dosimetric investigations for direct comparison of photon and proton dose degradation comprise only a small number of five,^20^ six,^21^ and ten^22^ patient datasets.

The presented in‐silico study investigates the difference of IMRT and IMPT plans for 31 advanced HNC patients at the time of treatment planning and after 4 weeks of radiochemotherapy by recalculating the plans on kilovoltage control CTs. The control CTs exhibit typical intratherapy changes which can be considered as a combination of day‐to‐day setup uncertainty and anatomical changes.

## 2 METHODS

### 2.A Patient data

A cohort of advanced HNC patients with UICC stage III or higher received PET/CT imaging prior and during definite radiochemotherapy in our clinic.^23^ For the presented retrospective planning study, CT images of the PET/CT scans taken before therapy (CT_plan_) and after approximately 20 fractions (CT_recalc_) were used from 31 patients without intubation and that were scanned both times with thermoplastic head‐and‐shoulder mask for a sufficiently large region in cranio‐caudal direction. Both CTs were acquired with the same protocol and have the same voxel size of either (1.37 × 1.37 × 5) mm^3^ or (0.98 × 0.98 × 3) mm^3^. Patient characteristics are summarized in Table 1. Patients gave their written consent and the local ethics committee approved the study.

**Table 1:** Patient characteristics and CTV sizes

ID	Tumor localization	Gender m/f	Age	TNM	UICC status	CTV_plan_/ml	CTV_recalc_/ml	ΔCTV/ml
1	Oral cavity (tongue)	m	54	T3N1	III	299.1	269.7	−29.4
2	Oral cavity (base of mouth)	m	63	T4N2c	IVb	532.0	486.6	−45.4
3	Oral cavity (tongue)	f	52	T3N2c	IVa	297.4	261.8	−35.6
4	Oral cavity (tongue)	m	45	T3N2c	IVa	329.0	290.8	−38.2
5	Oral cavity (tongue)	f	49	T3N1	III	295.7	285.1	−10.5
6	Oropharynx	m	56	T4N2b	IVa	299.3	244.2	−55.1
7	Oropharynx	m	64	T4bN0	IVb	395.6	303.8	−91.9
8	Oropharynx	m	55	T3N2c	IVa	311.1	278.8	−32.4
9	Oropharynx	m	65	T3N2c	IVa	263.6	243.0	−20.7
10	Oropharynx (base of tongue)	m	55	T4bN3	IVb	447.5	410.4	−37.1
11	Oropharynx	m	52	T4aN2c	IVa	499.3	471.9	−27.4
12	Oropharynx	f	54	T4N2c	IVa	260.7	245.9	−14.8
13	Oropharynx (base of tongue)	m	64	T3N2b	IVa	354.4	335.6	−18.8
14	Oropharynx	f	58	T4aN2b	IVa	237.5	224.6	−12.9
15	Oropharynx	m	54	T3N1	III	295.2	270.5	−24.7
16	Oropharynx	m	46	T4aN2b	IVa	289.1	262.0	−27.1
17	Oropharynx (base of tongue)	m	62	T4aN2c	IVa	311.7	296.0	−15.7
18	Oropharynx	m	47	T4aN2b	IVa	310.9	277.1	−33.8
19	Oropharynx (base of tongue)	m	53	T3N2a	IVa	513.8	455.8	−58.0
20	Oropharynx	m	59	T2N1	III	284.2	253.9	−30.3
21	Hypopharynx	m	67	T4aN3	IVb	833.8	735.1	−98.8
22	Hypopharynx	m	76	T3N0	III	244.7	233.1	−11.6
23	Hypopharynx	m	56	T4N0	IVa	375.1	340.0	−35.1
24	Hypopharynx	m	57	T4bN2a	IVb	394.0	295.2	−98.8
25	Hypopharynx	m	51	T3N2b	IVa	508.1	448.7	−59.3
26	Hypopharynx	m	52	T3N2b	IVa	299.5	261.0	−38.5
27	Hypopharynx	m	50	T4aN2b	IVa	473.6	401.7	−71.9
28	Hypopharynx	m	46	T3N1	III	209.4	202.8	−6.6
29	Hypopharynx	m	74	T4aN2b	IVa	325.9	306.1	−19.7
30	Larynx	m	69	T3N2c	IVa	319.0	287.1	−31.9
31	Larynx (epiglottis)	m	59	T3N2c	IVa	417.6	390.2	−27.4
	Median					311.7	287.1	−31.9

m, male; f, female; TNM, tumor, node, metastasis classification (all M0); UICC status, tumor classification according definition of Union Internationale Contre le Cancer; CTV_plan_, size of CTV contoured on CT_plan_; CTV_recalc_, size of CTV contoured on CT_recalc_; ΔCTV, CTV_recalc_ − CTV_plan_.

Targets and OARs were delineated on CT_plan_ as described earlier.^4^ The gross tumor volume (GTV) included the primary tumor and involved lymph nodes. The clinical target volume (CTV) was created by a 5–10 mm isotropic GTV expansion corrected for noninfiltrated bone and air cavities, and a prophylactic volume for elective lymph nodes defined according to Grégoire et al.^24^ was added. The planning target volume (PTV) was defined by CTV margins of 5 mm in cranio‐caudal direction and 4 mm in plane with a 3 mm distance to the external contour except for three patients with skin infiltration. A 10 mm build‐up bolus was applied for those patients to achieve adequate dose coverage in IMRT plans. The isotropic PTV concept was used for IMRT and IMPT planning for better comparability of dose distributions outside target volumes and same intersection of OARs with PTV. Published guidelines were applied for parotid gland^25^ and brachial plexus delineation^26^, ^27^ and internal guidelines for spinal cord, brain stem, and mandible delineation. Artifacts in soft tissue arising from metal implants were contoured and overwritten before dose calculation. Planning risk volumes with 3 mm margin for spinal cord, brain stem, and plexuses were included.

Contours were transferred from CT_plan_ to CT_recalc_ by deformable image registration and adjusted afterward. Workload of contour adjustment was split among two physicians. CTV sizes for both CT scans, and therefore a quantification of anatomical changes, are included in Table 1. Even though the CTs are taken from photon radiotherapy patients, the study assumes that anatomical changes induced by IMPT and IMRT are similar.

### 2.B Dose prescription, treatment planning

All dose values will be stated in Gy and refer either to absolute absorbed photon dose or to absorbed proton dose weighted by a constant relative biological effectiveness of 1.1. The intended treatment course consists of two series with homogeneous dose prescription of 2 Gy per fraction to the respective PTV. A full‐field series of 25 fractions (50 Gy) would be followed by a sequential boost series of 11 fractions (22 Gy; not evaluated in this study) to escalate the dose in the non‐elective target volume to 72 Gy. The aim was to irradiate at least 95% of the PTV with more than 95% of the prescribed dose (*V*_95%_ > 95%), to avoid dose levels above 107% (*V*_107%_ = 0%) and to provide a PTV *D*_mean_ close to prescription. OAR constraints for spinal cord (*D*_max_ < 45 Gy), brain stem (*D*_max_ < 54 Gy) and brachial plexus (*D*_max_ < 72 Gy) had higher priority than target coverage. Dose to parotid glands (*D*_median_ < 26 Gy) and mandible (minimum dose received by 1 ml: *D*_1ml_ < 75 Gy) was minimized without compromising target dose. In cases where the contralateral/ipsilateral parotid gland could not be assigned *a priori* due to the bilateral target volume, they were distinguished after treatment planning according to the lower/higher *D*_median_ value. Assuming equal dose contribution over 36 fractions, OAR constraints for the full‐field series of 25 fractions are *D*_max_ < 31.25 Gy, *D*_max_ < 37.5 Gy, *D*_max_ < 50 Gy, *D*_median_ < 18.06 Gy, and *D*_1ml_ < 52.08 Gy for spinal cord, brain stem, plexus, parotids, and mandible respectively. Due to the reduced boost target volume, the plexus constraint was slightly relaxed where intersecting the elective PTV.

For each patient, an IMRT and an IMPT plan for the full‐field series were calculated as described previously.^4^ Step‐and‐shot IMRT plans were optimized using the treatment planning system (TPS) Pinnacle^3^ (Philips Healthcare, Amsterdam, Netherlands) and consisted of seven almost equidistant but individually adjustable, coplanar 6 MV fields. Seven to nine fields are considered as optimal,^28^ but a prestudy (not published) revealed longer treatment times for nine fields without relevant dosimetric improvement. Aperture optimization was performed for a Siemens Artiste linear accelerator. Maximal 70 segments (each with dose ≥ 3 monitor units (MU) and size ≥ 3 cm^2^) were permitted.

IMPT plans were generated with multifield optimization in XiO^®^‐Proton (Elekta Instrument AB, Stockholm, Sweden). Unfortunately, this commercial and clinically used TPS has no possibility for robust planning, i.e. to account for potential uncertainties in patient positioning or range uncertainty during the optimization. Similar to other studies,^29^, ^30^ a 3‐field beam configuration was chosen. The IMPT plans had individually adjustable, coplanar beam angles: a left anterior oblique, a right anterior oblique and a posterior beam. Plans were calculated for an IBA universal nozzle with range shifter (7.4 cm water equivalent thickness), 5 cm air gap and Gaussian‐shaped pencil beams with nominal sigma beam width of 4 mm in air (highest energy, no range shifter). A spot distance of 4 mm and a minimum spot dose of 0.01 MU were used for optimization. Dose grid resolution was 3 × 3 × 3 mm^3^. Only regions inside the external contour were considered for dose optimization and calculation, i.e. everything outside the external contour was overwritten with air density to assess the pure influence of anatomical changes in the recalculation without interference of relative shifts of head pillow, mask mounting, and inhomogeneities of the PET/CT couch.

### 2.C Recalculation on CT_recalc_

**Figure 1:**
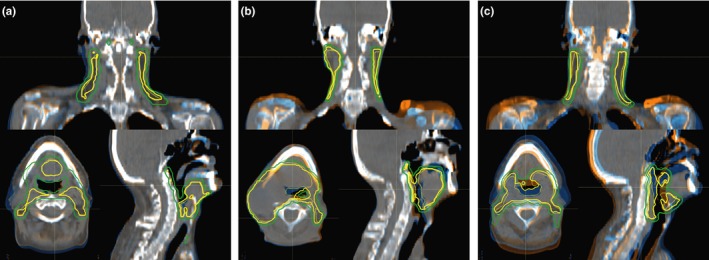
Examples for differently rated matching between CT_recalc_ (orange) and CT_plan_ (blue). Focus of attention was bony anatomy match; mainly vertebrae, shoulders, and mandible. Group I registration (a) showed acceptable match. Group II datasets (b) had considerable mismatch in certain regions like here for the shoulders. Group III (c) contains registrations for which a position adjustment is indicated since noticeable torsion and head tilts impede good matching by couch shifts and rotations. CTV (yellow) and PTV (green) are indicated as solid (CT_plan_) and dashed (CT_recalc_) contours.

The CT_recalc_ images were rigidly registered to the corresponding CT_plan_ utilizing a 6 degrees of freedom (DoF) algorithm (RayStation 4.5, Raysearch Laboratories AB, Stockholm, Sweden). The registration is based on gray values and the option to focus on bone (large Hounsfield units) match was applied. If results were suboptimal (13×), a manual 3 DoF mapping was performed with focus to upper vertebrae. The chosen registration was subjectively rated on its performance of bone match in the PTV region which is interpreted as setup error. To investigate the influence of setup uncertainty, a qualitative 3‐point scale was applied (Fig. 1) where I stands for acceptable positioning (13×), II for considerable mismatches of bone structures in certain areas (8×), and III for likely to be repositioned under clinical routine since noticeable torsion and head tilt impede good bone match throughout (10×). Transformation matrices were applied to CT_recalc_ via Plastimatch software. For plan recalculation in the respective TPS, same isocentre coordinates, beam angles, MU, dose grid, and CT to density conversion were applied.

Planned and recalculated dose distributions were analyzed with Computational Environment for Radiotherapy Research (CERR, v4.4) in MATLAB R2015a. Generated cold‐ and hotspots and differences in constraint‐relevant dose parameters for OARs were quantified, considering the near maximum dose *D*_1ml_ for spinal cord, brain stem, and plexus. Although the plans were optimized for PTV, the CTV is the relevant volume for which coverage and homogeneity needs to be fulfilled throughout therapy. Thus, CTV target coverage (*V*_95%_), overdose (*V*_107%_) and homogeneity index (HI = (*D*_2%_ − *D*_98%_)/*D*_p_)^31^ were evaluated, where *D*_2%_ and *D*_98%_ denote the minimum dose to 2% and 98% of the CTV and *D*_p_ is the prescribed dose. Conformity number with reference isodose (RI) of 95% was determined for the PTV as CN = (PTV_RI_/PTV)×(PTV_RI_/V_RI_),^32^ where PTV_RI_ denotes the volume of the PTV covered by the RI and V_RI_ the volume of the RI. For the healthy tissue outside the PTV, the integral dose (defined as the mean dose times the volume) and the size of hotspot volumes > 107% were calculated. Changes in dose parameters were tested for significance by two‐sided paired *t*‐tests including all 31 patients.

### 2.D Correlation between dosimetric changes, set‐up error, and anatomic change

Mann–Whitney *U* tests were performed to test for a causal relationship between lower CT matching quality (setup error) and tumor shrinkage (anatomic changes) and to test whether the observed dose degradations were correlated with rated CT matching quality. Only the clearly different matching groups I and III were included in these tests since group II assignment might be less reliable and could therefore conceal correlation. Spearman correlation coefficients (*r*_*s*_) were calculated for all 31 patients to examine correlation between dose parameter changes and the initial size or change of the CTV and PTV sizes after the first 20 fractions. If Mann–Whitney *U* tests indicated dependency of dosimetric changes from CT matching quality, Spearman correlation coefficients were calculated for group I data only (in order to eliminate the dependency from setup errors and to investigate whether target sizes or their changes influence directly the dose degradation in cases with sufficient patient setup). *P*‐values < 0.05 were considered as statistically significant. Due to bilateral target volumes, there was no clear geometric or dosimetric ipsi‐ and contralateral plexus. Thus, *D*_1ml_ values of either left or right plexus were considered for correlation tests, whichever showed stronger increase.

## 3 RESULTS

**Figure 2:**
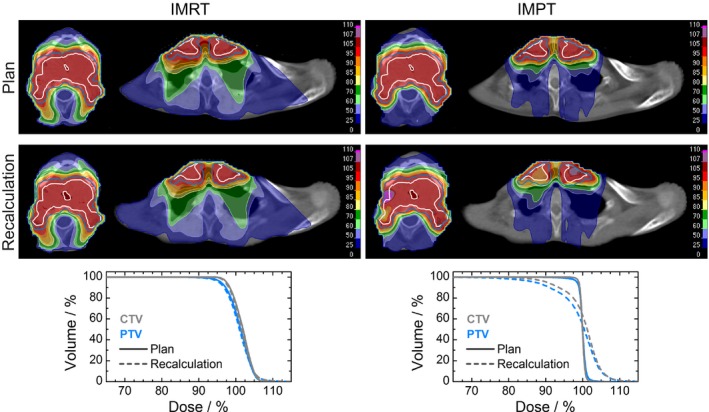
Two transversal slices of an exemplary planned and recalculated dose distribution on a CT dataset (group I matching) for IMRT (left) and IMPT (right) treatment plan. CTV (white contour, gray lines) and PTV (blue contour and lines) dose‐volume‐histograms demonstrate better conformity for IMPT in planned dose (solid) but stronger distortion in recalculated dose (dashed).

**Figure 3:**
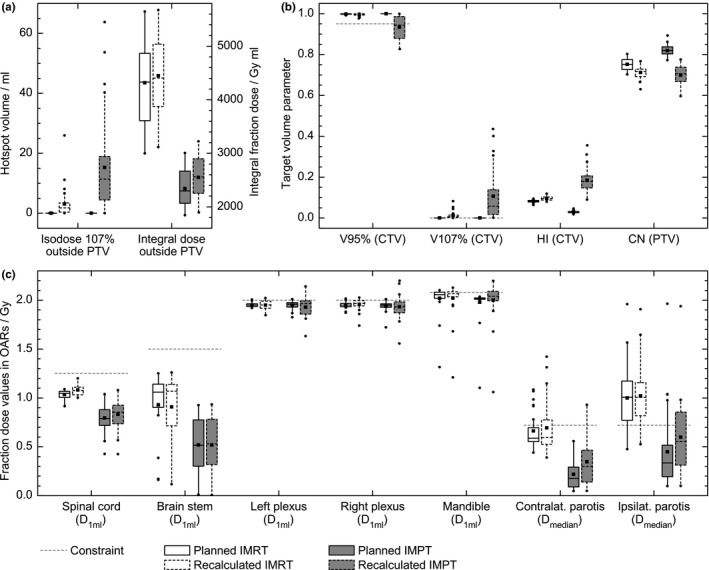
Statistics of investigated fraction dose values in healthy tissue outside PTV (a), in target volumes (b) and in OARs (c) for IMRT (white) and IMPT (gray) dose distributions as planned on CT_plan_ (solid outline) and recalculated on registered CT_recalc_ (dashed outline). Gray dashed horizontal lines indicate the respective constraints.

Exemplary dose distributions from IMRT and IMPT plans on CT_plan_ and CT_recalc_ are shown in Fig. 2. Statistics of investigated fraction dose parameters in OARs, targets and healthy tissue are presented in Fig. 3 and Table 2.

**Table 2:** Change of dose parameters for full‐field series of 25 fractions; median (range)

ROI parameter (constr.)	IMRT			IMPT		
	Plan	Recalc	Recalc‐plan	Plan	Recalc	Recalc‐plan
Organs at risk/healthy tissue outside PTV						
Spinal cord *D*_1ml_/Gy (<31.3)	26.2 (22.9–27.2)	26.9 (25.0–30.0)	0.9^a^ (−0.5–3.4)	19.7 (10.7–26.0)	21.4 (10.6–27.0)	0.2^a^ (−0.9–8.2)
Brain stem *D*_1ml_/Gy (<37.5)	26.5 (4.0–31.2)	26.7 (2.9–31.5)	0.4 (−18.1–2.3)	12.9 (0.3–23.1)	13.2 (0.2–23.3)	0.1 (−3.6–4.9)
Brachial plexus^c^ *D*_1ml_/Gy (<50.0)	48.9 (46.8–50.4)	49.4 (46.4–50.6)	0.6^a^ (−0.8–1.7)	49.0 (43.1–50.2)	49.4 (44.5–55.0)	0.5^b^ (−3.4–5.7)
Ipsilateral parotis *D*_median_/Gy (alap)	25.2 (11.9–49.0)	25.1 (13.1–47.7)	0.8 (−22.2–9.6)	8.4 (2.4–49.1)	13.8 (2.5–48.4)	3.4^a^ (−20.3–14.7)
Contralateral parotis *D*_1ml_/Gy (<18.1)	14.7 (11.0–27.1)	14.8 (9.7–35.5)	0.1 (−6.3–18.2)	4.4 (1.2–13.9)	7.5 (1.2–23.2)	1.3^a^ (−6.7–20.4)
Mandible *D*_1ml_/Gy (<52.1)	51.4 (32.9–52.5)	51.7 (30.2–53.2)	0.2 (−2.7–1.0)	50.4 (27.5–53.2)	51.0 (26.5–54.9)	0.8^b^ (−3.1–4.2)
External – PTV *V*_107%_/ml (→0.0)	0.0 (0.0–0.2)	1.9 (0.0–25.9)	1.8^a^ (0.0–25.9)	0.0 (0.0–0.0)	11.4 (0.0–63.8)	11.4^a^ (0.0–63.8)
External – PTV *D*_integral_/Gy·l (alap)	108.3 (74.9–141.2)	110.1 (77.9–141.8)	3.0^a^ (−6.6–9.0)	57.4 (46.1–75.1)	64.0 (47.4–80.6)	5.6^a^ (0.4–9.5)
Target volumes						
CTV V_95%_/%^d^ (>95.0)	99.9 (99.1–100.0)	99.7 (97.7–100.0)	−0.1^a^ (−2.0–−0.3)	100.0 (100.0–100.0)	94.4 (82.6–99.9)	−5.6^a^ (−17.4–−0.1)
CTV V_107%_/%^d^ (→0.0)	0.0 (0.0–0.2)	0.7 (0.0–8.2)	0.7^a^ (0.0–8.1)	0.0 (0.0–0.0)	5.8 (0.1–43.5)	5.8^a^ (0.1–43.5)
CTV HI (→0.0)	0.083 (0.064–0.095)	0.095 (0.080–0.119)	0.011^a^ (−0.001–0.033)	0.029 (0.018–0.046)	0.179 (0.090–0.355)	0.151^a^ (0.070–0.325)
PTV CN (→1.0)	0.752 (0.703–0.803)	0.717 (0.630–0.767)	−0.043^a^ (−0.084–−0.006)	0.820 (0.773–0.892)	0.706 (0.596–0.775)	−0.117^a^ (−0.223–−0.044)

constr., constraint; alap, as low as possible.

a Statistical significance (*P* < 0.05) for two‐sided paired *t*‐test.

b Statistical trend (*P* < 0.1) for two‐sided paired *t*‐test.

c Either left or right plexus considered, whichever showed larger Δ*D*_1ml._

d Differences are given in percentage points.

### 3.A Initial treatment plans

Planned dose distributions met planning objectives with CTV *V*_95%_ > 99% and *V*_107%_ < 0.2% and only slight exceedance of OAR constraints in some patients, mainly for IMRT parotids and mandible which were no dose limiting OARs. IMPT plans were superior (*P* < 0.001) in terms of CTV homogeneity, HI = 0.015 (0.009–0.023) (median, range), and PTV conformity, CN = 0.82 (0.77–0.89), in comparison to IMRT plans with HI = 0.041 (0.032–0.047) and CN = 0.75 (0.70–0.80). The healthy tissue outside PTV received no considerable hotspot doses (maximum *V*_107%_ < 0.2 ml). The median fraction integral dose was almost doubled in IMRT plans (4332 Gy×ml vs. 2298 Gy×ml).

### 3.B Changes after 20 fractions

The contoured CTV on CT_recalc_ was on average (37 ± 24) ml (mean ± standard deviation) smaller than on CT_plan_ (Table 1). PTV size decreased accordingly by about (131 ± 41) ml. Average parotid shrinkage was (4.1 ± 4.5) ml. Mann–Whitney *U* tests revealed no correlation between target volume changes and the CT matching score which rated the setup accuracy by the bone conformity. This shows that setup errors are not (necessarily) related to the anatomy changes quantified by target shrinkage.

Recalculated IMPT dose distributions were more inhomogeneous (Fig. 2), which was quantified by hot‐ and coldspot volumes > 1 ml. For recalculated IMPT plans, coldspots within the CTV of less than 90%, 80%, and 70% of the prescribed dose were observed in 20, 8, and 2 recalculated IMPT plans respectively. IMPT hotspots with doses above 110%, 115%, and 120% were found 23, 16, and 4 times inside CTV and 22, 11, and 5 times outside PTV respectively. In comparison, only 1 IMRT recalculation had a CTV coldspot below 90%; hotspots above 110% were observed only 5 times inside CTV and 6 times outside PTV and once above 115% outside PTV. In the context of reduced target volumes after 20 fractions, it is not surprising that the integral dose outside PTV increased significantly for both modalities, but it remained lower in IMPT cases [Table 2, Fig. 3(a)]. The initial advantage of IMPT plans was not preserved for target dose parameters [Table 2, Fig. 3(b)]: Conformity numbers decreased to values of about 0.7 for both modalities. The distributions of the CTV parameters *V*_95%_, *V*_107%_, and HI were inferior for IMPT. Especially the required coverage *V*_95%_ > 95% was not fulfilled anymore in 16 cases. The average HI increased by a factor of about 6 and the average *V*_107%_ by about 10 percentage points. Changes in IMRT target parameters were also statistically significant, but clearly less pronounced. Changes in OAR fraction dose parameters [Fig. 3(c)] were significant in spinal cord for both modalities, in plexus for IMRT and in both parotids for IMPT plans. Table 2 summarizes the parameter changes, whereas single fraction dose values were scaled to 25 fractions for a better rating of relevance. For example, median changes in spinal cord and plexus *D*_1ml_ were below 1 Gy for both modalities, while IMPT parotids *D*_median_ changes were considerably higher. Moreover, the maximum individual changes were always found for IMPT plans (up to several Gy), but IMPT OAR dose parameters for spinal cord, brain stem and parotids remained typically lower than those for IMRT.

### 3.C Correlation of dose degradation with CT matching quality, target size and shrinkage

Changes in investigated OAR dose parameters were not significantly different between sufficient (I) and less acceptable (III) CT matching quality for both modalities. For IMPT target dose parameters, change of CTV *V*_95%_ (*P* = 0.005), PTV CN (*P* = 0.001) and CTV HI (*P* = 0.004) was significantly worse for group III. This indicates that special effort is required for exact bone matching when positioning for proton therapy, especially when treating large elective HNC targets. Similar dependence for IMRT was only observed for PTV CN (*P* = 0.049). CTV *V*_107%_ was not correlated with CT matching quality (*P* > 0.3) for both modalities.

Moderate correlations (0.5 < |*r*_*s*_| < 0.6) between OAR dose parameter changes and initial size or shrinkage of target volumes was found for plexus *D*_1ml_ and *V*_107%_ outside PTV. More specifically, change in plexus *D*_1ml_ was correlated to initial CTV (*r*_*s*_ = 0.60), initial PTV (*r*_*s*_ = 0.57), change of CTV (*r*_*s*_ = −0.60) and change of PTV (*r*_*s*_ = −0.57) for IMPT and to PTV change (*r*_*s*_ = −0.51) for IMRT; the generation of healthy tissue hotspots was correlated to initial CTV (*r*_*s*_ = 0.52), initial PTV (*r*_*s*_ = 0.54) and change of PTV (*r*_*s*_ = −0.58) for IMPT. For the change of target dose parameters, only weak correlations (|*r*_*s*_| < 0.4) with target volumes were found. The only exception was the relation between PTV shrinkage and change of PTV conformity number (*r*_*s*_ = 0.67) in IMRT plans, i.e. anatomically changes would basically predict a loss of conformity, since planned fields would be too large for the shrunk target. A similar behavior was not observed for IMPT parameters.

## 4 DISCUSSION

We analyzed the potential dose degradation due to intra therapy changes for IMPT treatment plans in comparison to IMRT plans. Using for the first time a quite large cohort of 31 patients allowed for correlation analyses of dose distortion with CT matching, target size and shrinkage. Investigations for smaller numbers of patients (≤ 10) were performed recently^20^, ^21^, ^22^ utilizing 2–7 control CTs. Only one delineated intra therapy CT was available here. However, it was taken after about 4 weeks, a time point that is associated with the largest dose increase in some OARs for IMPT^22^ and that was used earlier for adaptation in IMPT HNC treatment with encouraging clinical outcome for less advanced HNC.^33^ Similar to previous studies, we found for IMRT that target dose parameters changed significantly but remained mostly within requirements while OAR dose increase was partly critical, e.g. for parotid glands. IMPT OAR dose changes had slightly larger diversity but remained mostly well below constraints since they were already lower in the initial plan. A substantial loss of target coverage, formation of hotspots and loss of homogeneity and conformity was observed for IMPT which is in accordance with Müller et al.^20^ and Góra et al.^21^. However, Thomson et al.^22^ showed no worsening of target doses for IMPT investigating 10 oropharynx cancer patients with similar disease stage as in our cohort. In their study, nodal level IV was excluded from contouring, since it was not covered by their cone‐beam CT images, i.e. shoulders were excluded as well, but shoulder mismatch was found to be a typical issue in our patients. Furthermore, their generated IMPT plans can be considered as more robust since a mixture of single‐field and multi‐field optimization was applied which was not feasible with our TPS. The robustness of conventional IMPT plans, like ours, is lower since each beam is allowed to treat the complete CTV, which might especially be problematic for the posterior beam and the anterior target portions in the lower neck. Further advanced planning approaches^34^, ^35^, ^36^ and, in particular, robustness analyses^36^, ^37^, ^38^, ^39^, ^40^ need to be considered for future studies and to be translated into clinical practice. So far, there is no uniform consensus about the ideal IMPT planning strategy for HNC patients and robust optimization cannot be considered as clinical standard yet. Today, several commercial TPSs provide the possibility for IMPT planning but often without (enough) dedicated tools and algorithms to address the proton‐specific problems. Our IMPT plans may be inferior compared to the best achievable on the market, but, to the best of our knowledge, they are not unrealistic. Therefore, the results of this study with the clear IMPT dose degradation in the control CT should be understood as an appeal to make any effort to ensure safe and reliable proton therapy delivery.

Although inter‐observer variability in contouring might be of concern for our study (two physicians involved), we applied the same contours for IMRT and IMPT and the overall different influence of setup errors and anatomy changes for both modalities became apparent: If the investigated patients had been treated with IMPT, an adaptive replanning would have been indicated at the latest after 4 weeks of therapy for about half of the cohort, solely based on the unacceptably decreased target coverage. Since not all potential errors like e.g. systematic range errors due to CT calibration uncertainty were considered in this study, the results could even be worse in clinical practice.

However, it can be concluded that IMPT treatment plan adaptations will be driven by dose degradation in target volumes. IMPT dose parameters for OARs remained typically below the clinical constraints and their degradations were neither correlated with scored CT matching nor with initial target size or target shrinkage, except for the brachial plexus and the hotspot formation in healthy tissue. Since change of target volumes were not correlated with maintaining of target coverage, homogeneity and conformity even for accurate positioning, those measures are probably not predictable from observed anatomical changes and IMPT dose recalculation is required. Kraan et al.^41^ reported correlation between increased CTV *V*_107%_ and initial CTV size, which was low in our study containing more advanced HNC, but moderate correlation between PTV size/shrinkage and formation of hotspots in healthy tissue was found here.

Besides indicating the importance of monitoring anatomic changes and performing plan adaptation, we have shown that reasonable effort is required for exact patient positioning, since loss of target coverage, homogeneity and conformity were significantly worse for less accurate CT matching for proton plans. Shoulder adjustment and verification of head tilt under the mask system is essential; repositioning by couch shift only is insufficient. We believe that the suboptimal CT matching in this study is realistic for current radiotherapy treatment. The standard image guidance for positioning in proton therapy is orthogonal X‐ray^42^ which has limitations for 3D target positioning. More advanced 3D imaging techniques like in‐room and cone‐beam CT become more and more available and might be beneficial,^43^, ^44^, ^45^ also for direct dose recalculations, dose accumulation and treatment adaptations. Estimations for accumulated doses during the treatment course were not feasible here due to the limitation of having only one control CT available. Thus, no conclusions could be drawn for impact of anatomical changes on biological endpoints like normal tissue complication and tumor control probability, and no investigations on optimal adaptation time points could be performed.

## 5 CONCLUSION

IMPT plans provide superior dose distributions in advanced HNC but these are more prone to intra therapy changes. The study underlines that precise positioning and monitoring of anatomy changes are mandatory for reliable IMPT treatment. In consideration of the larger absolute changes, IMPT plans might require adaptation more often than IMRT plans. Since OAR doses remained typically below constraints, indications for adaptive IMPT should rather be derived from target dose degradation.

## CONFLICT OF INTEREST

The authors declare no conflict of interest.
